# Increasing robotic rectal cancer surgery may not increase total hospital costs

**DOI:** 10.1007/s11701-025-02921-1

**Published:** 2025-11-08

**Authors:** Jukka Rintala, Johanna Mäkelä-Kaikkonen, Pasi Ohtonen, Elisa Mäkäräinen, Tero Rautio

**Affiliations:** 1 Medical Research Center Oulu, Oulu University Hospital, Oulu, Finland; 2 PL29, Oulu, 90029 OYS Finland

**Keywords:** Robotic, Laparoscopic, Rectal resection, Rectal cancer, Cost analysis

## Abstract

**Background:**

The robotic approach provides fewer conversions and complications as well as better post-operative functionality in rectal surgery but increased costs in randomized trials has limited its use. The aim of this study was to compare the total hospital costs of rectal cancer surgery following the introduction of a second robotic platform.

**Methods:**

Consecutive patients who underwent open, laparoscopic or robotic rectal cancer resection between March 2020 and February 2023 were retrospectively collected from patient registry. Time periods represented 18 months before and 18 months after the introduction of second robotic system. Outcomes and 90-day hospital costs of 3/2020-8/2021 (Group1) and 9/2021-2/2023 (Group2) were compared.

**Results:**

Total cohort included 227 patients, 105 in Group1 and 122 in Group2. No significant differences were observed in ASA-class, Tumor-location, -stage or co-morbidity. Operative blood loss and operative time were similar. Increased robotic access changed the allocation of surgical modalities (*p* < 0.001) and increased the share of patients operated mini-invasively (83% vs. 93%, *p* = 0.038), also major complications decreased (48% vs. 37%, *p* = 0.041). Mean operative costs were higher in Group2 (13 350 vs. 12 819 €) but mean 90-d total hospital costs were lower (23 777 vs. 24 875 €, non-significant).

**Conclusion:**

Increased robotic access changed the allocation of surgical modalities in rectal cancer surgery. Although, new robotic platform increased direct operative costs, total 90-d hospital costs did not rise due to compensatory decreases in other segments of the treatment. Therefore, increasing robotic surgery may not increase total hospital costs of rectal cancer surgery.

## Introduction

Minimally invasive rectal resections have been extensively studied in the past decade, demonstrating safety and efficacy profiles comparable with open surgery, with notable improvements in early postoperative recovery having potential economic advantages. Due to the inherent limitations of laparoscopic technique, narrow pelvic operations, and high requirements for assistants, laparoscopic rectal cancer surgery is difficult to master, and learning curve for laparoscopic rectal cancer surgery is longer and easily results in a high conversion rate and postoperative complications in the learning phase [1–3].

Robotic surgery for the treatment of rectal cancer is an emerging technique that can overcome some of the technical drawbacks posed by conventional laparoscopic approaches, improving the scope and effect of radical operations. Recent studies have shown that robotic rectal cancer surgery has emerged as a promising alternative, providing benefits such as improved precision, reduced invasiveness, and better outcomes in terms of fewer conversions, complications, reoperations and better postoperative functional symptoms [4, 5]. Also, the controlled learning curve of robotic techniques has been proven to be safe [6]. Therefore, robotic assisted procedures have become increasingly commonplace and the ease of use with the da Vinci robot has led to widespread acceptance among colorectal surgeons [7].

Several studies have compared a large series of robotic and laparoscopic surgical approaches and assessed short and long-term oncological outcomes without any major clinical or statistical differences [8–10]. However, according to the results of recently published largest RCT from China showed significantly improved long-term oncological outcomes in patients with middle or low rectal cancer operated using robotic platform [11]. While randomized controlled trials and observational studies have verified the safety and effectiveness of robotic surgery in specialized centers, they rarely assess its economic impact on healthcare [12]. Only a few studies in literature address the cost issue of the robotic system in surgery [13, 14].

Korean review article concluded that almost all studies published from 2012 to 2018 suggested that robotic TME had definitely higher costs than laparoscopic surgery, while the overall clinical outcomes were similar [15]. This has created persistent arguments against robotic surgery, although these results are from early era of robotic surgery, when old fashioned clumsy robotic systems with limited range were used without modern instrumentation needed in rectal cancer surgery. Additionally, most of the reviewed studies compared only operative costs of robotic and laparoscopic surgery for rectal cancer, which are still higher because of the expensive purchasing charge for the robotic systems.

Spanish research group found less clear difference in overall costs, however favouring robotic surgery. Their total cost of the rectal cancer surgery was surprising low compared to expenses reported in this study [15]. Recently published retrospective, case-matched patient cohort study was not powered to detect a significant increase in cost with robotic abdominoperineal resections [16]. However, by utilization of the robotic system APR technique can be tailored consist of patient and tumor characteristics instead of using routinely ELAPE type of ultraradical procedures needing the jack knife position and perineal reconstruction.

The aim of this study was to assess the cost-effectiveness of increased robotic rectal cancer surgery by comparing two time periods before and after investment on robotic systems while reckoning also possible changes in the allocation of different surgical modalities.

## Materials and methods

### Study design

This is an observational, retrospective cross-sectional study using a prospectively developed database and analyzing the outcomes of patients who underwent rectal cancer surgery between 3/2020 and 2/2023. The retrospective design was chosen to utilize pre-existing clinical registry data while ensuring patient confidentiality through pseudonymization.

### Patients

All consecutive patients who underwent open, laparoscopic or robotic-assisted anterior resection (AR), abdominoperineal resection (APR) or Hartmann’s procedure for primary rectal adenocarcinoma at Oulu University Hospital between March 2020 and February 2023 were retrospectively evaluated. Exclusion criteria included T4b tumors requiring multi-organ resection, rectal malignancy other than adenocarcinoma, emergency procedures, planned rectal surgery with major concomitant procedures, and metastatic disease without the possibility of curative surgery. Data on demographic, perioperative and short-term outcomes were collected retrospectively from prospectively collected registry file. Additional data needed for analysis were retrieved from the patients’ medical records.

### Study setting and context

The study was conducted at the tertiary cancer center Oulu University Hospital, where robotic rectal cancer surgery has been performed since 2011 using Da Vinci Si robotic system. Second, Da Vinci robotic system Xi was available on September 2021 increasing robotic OR capacity and Si system was replaced by X system during next year. All procedures followed a standardized intraoperative sequence, performed by a single surgical team consisting of six experienced colorectal surgeons, ensuring consistency in surgical techniques across all cases.

### Surgical procedures

Operative planning was made in MTD meetings following national rectal cancer treatment guidelines. Surgical techniques were chosen patient-by-patient and surgeries followed the principles of total mesorectal excision. Rectal resections were performed in accordance with each surgeon’s usual practice, but open, laparoscopic and robotic operations have been all standardized (e.g. used instruments, portsite placement, mobilisation of the splenic flexure, inferior mesenteric artery/vein division, high versus low vascular division etc.). The Rectal cancer surgical team had six surgeons and four of those performed all robotic operations. Experienced colorectal surgeons performed robotic operations using the DaVinci surgical systems Si, X or Xi according to standard protocols of each robotic system. Robotic-assisted surgery involved always a totally robotic approach including splenic flexure mobilization. Conversion to open operation was defined as the use of a laparotomy wound for any part of the mini-invasive surgery.

### Variables and data collection

Preoperative data included demographic characteristics, clinical history, laboratory results, and imaging findings, ensuring a comprehensive patient assessment. The comorbidity was assessed according to surgical Charlson Comorbidity Index (CCI) (PMID: 20306528). Intraoperative data, such as operative time, surgical approach, and intraoperative complications, were systematically documented in surgical reports. Postoperative data were extracted from discharge summaries and follow-up clinic records, ensuring accuracy through cross-referencing electronic medical records. All data were digitally stored, guaranteeing confidentiality and structured data management. The primary postoperative outcomes included: length of hospitalization, procedure-related complications, such as hematomas, infections, and anastomotic leaks and other related postoperative events graded using the Clavien-Dindo classification of surgical complications (PMID: 15273542). Post-operative data also covered outpatient clinic visits of the first 90 days and possible re-admissions during this period.

### Hospital cost analysis

Hospital costs for the study cohort were obtained from the OUH financial department to assess the actual costs incurred by individual patients. In our hospital system, operative costs are based on aggregated activity-based costing analyses of procedural codes of the Nordic Medico-Statistical committee. All surgical procedures have their individual code and the operative costs in OUH are based on the main operative code in those cases that multiple procedures are made within the same operation. However, robotic operations have their own pricing code including acquisition, instrumental and maintenance costs for the Da Vinci robotic systems, which is added to the price of main procedure when robot is used in the operation. Other components of the treatment (e.g., hospital stay, laboratory tests, radiological imaging) are priced individually according to the patient´s resource utilization.

Our cost analysis included all hospital-related expenses incurred within 90 days following the primary surgery, excluding any elective treatments unrelated to rectal cancer that were scheduled prior to the initial rectal cancer operation. Additionally, oncological treatment costs were excluded, as they are determined primarily by cancer stage rather than surgical modality or postoperative complications.

Therefore, the cost analysis encompassed the primary hospitalization period (including the index operation, inpatient stay, laboratory and radiological investigations, potential intensive care, and reoperation costs), any readmission costs, and postoperative outpatient clinic visits.

All reported costs reflect actual patient-level expenditures and not the charges billed to payors. Costs were adjusted for inflation using the Statistics Finland Consumer Price Index, and are presented in Euros, standardized to the 2025 price level.

### Follow-up and data completeness

All patients were seen in the outpatient clinic for follow-up evaluations at 1 month, 6 months, and 12 months, no patients were lost to follow-up, ensuring a complete dataset for analysis.

### Bias minimization

To ensure methodological consistency and reduce potential biases we have had consistent surgical team and standardized protocol for operative treatment of rectal cancer. The same group of experienced surgeons performed all procedures, ensuring uniformity in surgical techniques and decision-making. A unified preoperative, intraoperative, and postoperative protocol was followed during the study period.

### Statistical methods

Descriptive statistics were used to summarize the data. Categorical variables were compared with chi-squered test and continuous variables were compared with Mann-Whitney U test due to their non-normal distribution. To adjust the impact of study period on total costs for confounding factors, two multivariable adjusted linear regression models were used: model 1 (confounding factors: tumor distance from anal margin, radiological T-stage, ASA-class, CCI, Age) and model 2 (all confounding factors in model 1 and preoperative radiation therapy). Regression coefficients for period effect with 95% confidence intervals are presented as the result of the regression models. Two-tailed P of less than 0.05 was considered statistically significant. Statistical analyses were conducted by using IBM SPSS software version 29.0. Patients with missing values were excluded from the analysis of that variable.

### Limitations

This study has inherent limitations primarily associated with its retrospective design, which may introduce potential biases, including selection and reporting bias. Selection bias may arise from the inclusion of patients who met specific institutional criteria, which could limit the external validity of our findings. Additionally, reporting bias may result from variations in documentation practices and differences in the completeness of medical records. Nevertheless, the use of a previously established protocol for data collection helped mitigate these concerns by ensuring greater uniformity and consistency in the information gathered. We used OUH hospital costs/prices that are based on internal accounting of mean costs of operations on procedural codes of the Nordic Medico-Statistical committee and mean other cost segments in our hospital. These hospital costs may differ from other hospitals and/or health care systems. However, internal accounting of hospital prices is and should be evaluating local financial conditions when composing hospital price levels. Also, we compare the prices of two different time periods within the same hospital environment which decreases the importance of nominal costs.

### Ethics approval

The study was conducted in accordance with the principles outlined in the Declaration of Helsinki. Since this was a retrospective analysis using anonymized data obtained during routine clinical care, formal ethics committee approval was not required under institutional and national research guidelines. Data confidentiality and privacy were strictly maintained throughout the study.

## Results

### Surgical modalities

Originally, the data of 227 patients were collected. After dividing the patients according to date of operation, a total of 105 patients for group 1 and 122 patients for group 2 were included in the analyses. The groups did not differ in terms of the baseline characteristics presented in Table 1. There was a minor difference in surgical techniques, naturally robotic operations were more common in group 2; 47/105 patients (45%) vs. 93/122 (76%) (Table 2). Less APRs, 50 operations (48%) vs. 53 (43%) (p 0.38) and more anastomoses 63 (52%) vs. 49 (47%) (*p* = 0.51) were done during the later period as seen in Table 2 but these differences were non-significant. Conversion percentage in laparoscopic procedures was 13% in both groups and there was only one conversion during robotic operation. However, due to the changes in preoperatively chosen surgical technique, the share of mini-invasively operated patients increased from 87 patients (83%) to 113 patients (93%, *p* = 0.038). Patient allocation changed within modalities: in Group1, 21 laparoscopic operations (53%) were APRs and 19 (47%) ARs. However, in Group2, 6 laparoscopic ARs were performed (29% of laparoscopic operations) while that of 15 APRs (71%). Simultaneously, robotic APRs increased from 19 to 32 operations, but their share of robotic resections decreased from 40% to 35%. Therefore, more ARs was operated with robotic approach in Group2, 60 operations (87%) than in Group1 (28 operations, 51%).

**Table 1:** Baseline characteristics

Preoperative	Group1 *n* = 105	Group2 *n* = 122	*P* value
Age, Y, mean (SD)	66 (12.07)	67 (10.29)	0.40
Sex			0.40
Male Female	67 (64) 38 (36)	85 (70) 37 (30)	
BMI, mean (SD)	27.24 (5.7)	26.65 (5.2)	0.58
ASA score			0.71
I-II III IV	61 (58) 34 (32) 10 (10)	74 (61) 40 (33) 8 (7)	
CCI			0.59
0 1 2 or more	61 (58) 29 (28) 15 (14)	64 (53) 35 (29) 23 (19)	
Tumor distance from anal margin			0.661
High 12–18 Intermediate 6–12 Low < 6 cm	19 (18) 27 (26) 59 (56)	18 (15) 37 (30) 67 (55)	
cT			0.96
T1 T2 T3 T4	26 (25) 22 (21) 43 (41) 10 (10) Missing 4	23 (19) 21 (17) 61 (50) 14 (12) Missing 1	
cN			0.14
N0-nx N1 N2 Metastases Neoadjuvant radiotherapy	44 (42) 25 (24) 32 (30) 2 (2) 43 (41)	53 (43) 42 (34) 26 (21) 5 (4) 57 (47)	

CCI: Charlson Comorbidity Index

**Table 2:** Intraoperative data

	Group1 *n* = 105	Group2 *n* = 122	*P* value
Surgical time, min, mean (SD)	312 (109)	311 (87)	0.64
Technique			< 0.001
Open surgery Laparoscopic Robotic-assisted	12 (11) 46 (44) 47 (45)	5 (4) 24 (20) 93 (76)	
Anterior resection Abdominoperineal resection Hartmann’s Rectal anastomosis	55 (52) 50 (48) 6 (6) 49 (47)	69 (57) 53 (43) 6 (5) 63 (52)	0.38 0.51
Overall conversion Conversion from laparoscopy (conversion-%) Conversion from robotic (conversion-%)	6 (6) 6 (13) 0 (0)	4 (3) 3 (13) 1 (1)	0.34
Mini-invasively operated	87 (83)	113 (93)	0.038
Blood loss, cc, mean (SD)	232 (322)	231 (424)	0.30

### Short term outcomes and post-operative complications

Table 3 presents the postoperative data showing equal oncological results, no changes were observed in the numbers of R0-resections 100 (95%) vs. 118 (97%, *p* = 0.66) or harvested lymph nodes (mean 23 vs. 22, *p* = 0.93). In overall complication rate, no significant difference was observed but increasing of robotic approach decreased major complications (CD III-V, 50 (48%) vs. 41 (37%), *p* = 0.041). Median in-hospital stay (9,0 days vs. 7,0 days, *p* = 0.37) was shorter in the group 2 but the difference was non-significant. However, patient operated with AR had shorter hospital stay in Group2 (5 vs. 7 d, *p* = 0.015). No significant changes in hospital stay were observed within surgical modalities between time periods (Table 4).

**Table 3:** Post-operative data

	Group1 *n* = 105	Group2 *n* = 122	*P* value
Comp. Comp. Ind.	19.89 (17.3)	16.07 (15.6)	0.20
Anastomotic leak			0.64
No (%) Requiring surgery	5 (10) 3 (6)	4 (6) 3 (5)	
Clavien-Dindo			0.21
No complications I II IIIa IIIb IV V Major complications (CD III-V)	34 (32) 8 (8) 13 (12) 24 (23) 25 (24) 0 (0) 1 (1) 50 (48)	51 (42) 7 (6) 23 (19) 17 (14) 23 (19) 1 (1) 0 (0) 41 (37)	0.041
Readmission	24 (23)	28 (23)	0.99
pT			0.79
tx T1 T2 T3 T4	2 (2) 9 (9) 32 (31) 57 (55) 3 (3)	10 (8) 14 (12) 46 (38) 50 (41) 2 (2)	
pN			0.80
N0 N1 N2	69 (66) 25 (24) 11 (10)	77 (63) 33 (27) 12 (10)	
Resection			0.66
R0 R1	100 (95) 5 (5)	118 (97) 4 (3)	
Lymph nodes, mean (SD) Med.	23 (15) 19	22 (17) 21	0.93
Hospital stay, d, mean (SD) Med.	9.9 (5.6) 9	8.8 (6.9) 7	0.37
Hospital stay, d, med, (mean) [SD]			
AR	7, (8.4), [5.1]	5, (7.2), [8.4]	0.015
APR	10, (11.5), [5.6]	10, (10.5), [4.1]	0.49
Hartmann’s	10.5, (10.2), [4.1]	9, (11.0), [5.7]	0.94

**Table 4:** Post-operative hospital stay before and after new robotic platform according to surgical modality

	Group1 *n* = 105	Group2 *n* = 122	*P* value
Open surgery N	**12**	**5**	
Hospital stay, mean (med)	11.5 (11)	11.0 (9)	0.72
Laparoscopy N	**40**	**21**	
Hospital stay, mean (med)	9.5 (8)	9.3 (7)	0.80
Robotic N	**47**	**92**	
Hospital stay, mean (med)	9.2 (8)	8.4 (7)	0.12
Conversion from Lap. N	**6**	**3**	
Hospital stay, mean (med)	14.5 (9)	14.7 (14)	0.55
Conversion from Rob. N		**1**	
Hospital stay, mean (med)	−	7 (7)	−

Bold values designate surgical modality

### Cost analysis

The operative costs of anterior resections and abdominoperineal resections with different surgical modalities are presented in Table 5. The mean operative cost of laparoscopic AR was decreased in Group 2 (*p* = 0.04) but otherwise no significant changes within operative costs was seen between time periods. During the whole study period, the mean operative costs of robotic AR were significantly higher (11 869 € *n* = 88) than that of laparoscopic AR (9391 €, *n* = 25, difference 2478 €, *p* < 0.001). APR was also more expensive in robotics (15 282 €, *n* = 51) than in laparoscopy (12 959 €, *n* = 35, difference 2323 €, *p* < 0.001). The combined difference in operative price between robotic and laparoscopic rectal resection, disregarding operation type (AR or APR), during the study period was 1784 € (13 214 − 11 430 €). This difference was further decreased in intention-to-treat analysis to 1288 € (13 200−11 912 €) due to changes in conversion rates.

**Table 5:** Operative costs during primary hospital stay

	Group1 *n* = 105	Group2 *n* = 122	*P* value	All (mean)
**Open surgery**	**15 806** **(9203**,** 49 937)[11 047] n = 12**	**20 344** **(9375**,** 48 109)[15 750] n = 5**		**17 141**
AR	9378 (9202, 9749)[250] *n* = 4	9375 *n* = 1	n.a.	9289
APR	19 202 (13 339, 49 937)[12 503] *n* = 8	23 085 (12 851, 48 108)[16 752] *n* = 4	0.86	20 375
**Laparoscopy**	**11 275** **(7373**,** 15 594)[2271] n = 40**	**11 738** **(8182**,** 13 723)[2124] n = 21**		**11 430**
AR	9500 (7372, 14784)[1815] *n* = 19	8616 (8182, 8844)[260] *n* = 6	**0.04**	9391
APR	12 881 (11 556, 15 593)[1176] *n* = 21	13 075 (12 705, 13 722)[377] *n* = 15	0.59	12 959
**Robotic**	**13 050** **(8919**,** 19 686)[2483] n = 47**	**13 298** **(9391**,** 41 461)[3906] n = 92**		**13 214**
AR	11 489 (8919, 17 513)[1488] *n* = 28	12 261 (9391, 41 460)[4421] *n* = 60	0.57	11 869
APR	15 350 (13 933, 19 686)[1745] *n* = 19	15 240 (11 674, 19 599)[1270] *n* = 32	0.38	15 282
**Conversion from** **Laparoscopy**	**15 314** **(8222**,** 30 368)[9933] n = 6**	**14 753** **(9313**,** 19 801)[5255] n = 3**		**15 127**
AR	8984 (8222, 9667)[609] *n* = 4	9313 *n* = 1	n.a.	8765
APR	27 973 (25 579, 30 367)[3386] *n* = 2	17 473 (15 145, 19 800)[3292] *n* = 2	n.a.	22 273
**Conversion from** **Robotic**		**11 220** *n* = 1		**11 220**
AR	–	11 220 *n* = 1	–	11 220
APR	–		–	

Presented as: € mean (min - max), [SD]

Bold values designate surgical modality

The total hospital costs are presented in Table 6. When all patients were included, the introduction of a new robotic platform increased mean operative costs by 531 € (from 12 819 to 13 350 €). However, all other cost segments (mean hospitalization, re-admission, laboratory, radiological, out-patient clinic and other hospital costs, Table 6) were lower after the increased robotic access. These differences were statistically non-significant. Total 90-day hospital costs decreased after the introduction of a new robotic platform by 1098 € (from 24 875 € to 23 777 €, difference 1098 €, *p* = 0.23). When total hospital costs were adjusted for confounding factors, the cost decrease was diminished to 904 € in model 1 (95% CI -3854–2047, *p* = 0.55) and to 811 € (95% CI -3671–2048, *p* = 0.56) in model 2.

**Table 6:** Cost analysis including total hospital costs and its components

	Group1 *n* = 105	Group2 *n* = 122	*P* value
Total hospital costs	24 875 (12 746−76 074), [10 970]	23 777 (12 226−88 696), [11 124]	0.23
Operative cost	12 819 (7372–49 938), [4964]	13 350 (8182–48 109), [4855]	0.21
Hospitalization costs	6761 (1996–22 617), [3658]	5893 (1270–39 373), [4342]	0.17
Re-admission costs	2554 (0–35 910), [6760]	2117 (0–21 633), [4664]	0.79
Laboratory costs	812 (85–2508), [500]	746 (68, 4090), [562]	0.15
Radiological costs	510 (0–3125), [620]	500 (0–2825), [551]	0.89
Post-operative out-patient clinic costs	1177 (0–18 918), [2134]	982 (0–15 103), [1490]	0.85
Other hospital costs	241 (0–6195), [799]	188 (0–6980), [809]	0.57

Presented as: € mean (min-max), [SD]. Other Hospital costs include: consultation fees, special drug costs, intensive care costs, etc

## Discussion

This study demonstrates that robotic rectal cancer surgery may not be more expensive than traditional operative treatment of rectal cancer when total costs and all operated patients are calculated. Increased robotic access changed patient allocation between surgical modalities and increased the share of patients operated mini-invasively. We could also show that robotic surgery does not take more OR time in experienced robotic centre. In addition, increased robotic access decreased major complications (Clavien dindo III-V).

At the institutional level, the initial investment and ongoing maintenance costs associated with robotic systems are substantial, raising concerns about cost effectiveness and resource allocation in healthcare systems. These investments are usually covered by the hospitals and the departments executing rectal cancer surgery within their semi-fixed budgets. Typically, the administrative focus is on the direct costs of robotic platforms rather than on the total cost of the treatment. Although there may be overall economic gains over time, the initial costs and the prevailing silo mentality of departmentalized budgets often dominate shared decision-making when considering expensive investments. Furthermore, only minority of investment decisions in health care are based on scientific studies of cost effects. However, investments on surgical infrastructure should be addressed regarding their effect on the total hospital costs.

We show here that increasing access to robotic platforms alters patient allocation, allowing robotic surgery to partially replace open surgery rather than just laparoscopic procedures. Together with decreased number of open surgery and decreased number of conversions from laparoscopy, the share of mini-invasively operated patients increased. This decreases in part total hospital costs by enabling faster recovery and thus decreasing hospitalization. In addition, we show here that increased robotic access decreased major post-operative complications which also contributes to the compensation of increased operative costs of robotic surgery. Therefore, total hospital costs decreased following the increased use of robotic surgery. This effect on 90-day total hospital costs was not statistically significant and may thus be attributable to confounding factors. Nevertheless, our findings suggest that the significantly higher operative costs associated with robotic surgery were offset by reductions in ward costs, readmission costs, and postoperative outpatient clinic expenses. In part, these reduced costs may be due to minor, non-significant, changes in the increased proportion of rectal anastomoses (anterior resections) compared to abdominoperineal resections, the decreased rate of postoperative complications, and decrease in postoperative hospital stay which we observed after the increase in our robotic access. While these individual cost savings were modest, they underscore the importance of monitoring all components of hospital expenditure when evaluating the economic implications of surgical technologies and infrastructure.

The current clinical selection process for rectal cancer patients involves choosing among three surgical approaches: open surgery, laparoscopic surgery, and robotic surgery. Within this clinical decision-making process, patients undergo the surgical technique available that is deemed most appropriate for them. However, access to robotic platform is often limited, which restricts its broader use. Therefore, in clinical setting, surgeons typically first allocate into the robotic platform patients considered most likely to benefit from robotic approach. This may lead to a skewed selection where more challenging patients are operated with robotics. In randomized trials robotic and laparoscopic techniques are considered substitutive. However, in clinical practice, the alternative for robotic rectal resection may not always be similar laparoscopic operation. The robotic approach may facilitate mini-invasive rectal anastomosis in borderline cases particularly in patients with a narrow pelvis and/or low rectal tumors. Furthermore, some technically challenging patients may be eligible for minimally invasive surgery only through the robotic approach. As a result, cost comparisons between laparoscopic and robotic approach in rectal surgery may be misleading both in randomized and in non-randomized setting due to selection bias. According to our results operative cost difference between robotic and laparoscopic rectal resection was 2478 € when comparing similar operations with different modalities, 1784 € when comparing end results of different modalities after clinical patient allocation and 1288 € if one compared intention-to-treat costs of that clinical allocation. From the institutional point of view, the cost increase was 531 € when all rectal cancer patients were included. These increases were compensated with cost reduction in other segments of treatment. Therefore, the cost effect of increased robotic surgery can be evaluated from multiple points of view during the treatment and one should emphasize whether the aim is to evaluate the costs from patients or institutions aspect.

We observed that during the study period, mean total cost of laparoscopic rectal resection was in fact higher than robotic resection which contradicts the results of most randomized trials. Our finding is due to patient allocation of more expensive abdominoperineal resections. Before increased robotic access, 48% of laparoscopic resections and 40% of robotic resections were abdominoperineal resections and this share was increased in laparoscopy to 71% after new robotic platform was introduced but decreased to 35% in robotic operations. These changes skewed distribution of patients between modalities even more and were done probably because surgeons allocated more resections with anastomoses to robotics. These results indicate that outside randomized trials laparoscopy and robotic platforms are not purely substitutive tools but should be evaluated as complementary.

In order to robotic rectal surgery to further develop the field of minimally invasive surgery, there should be an obvious cost-effective advantage over laparoscopic surgery. Therefore, it is crucial that large-scale prospective trials are performed to address the cost-efficiency of robotic rectal surgery. However, part of the overall cost effect of robotic surgery appears to become from replacing open surgery which should be included in the cost analysis. Furthermore, goal should be to solve the actual cost-effect of robotic platform on the costs of rectal cancer treatment instead of only the costs of those patients operated with robotic approach. In our unit, increasing robotic rectal surgery increased the share of mini-invasively treated patients, decreased major complications and did not increase total hospital costs. This indicates that in clinical setting, robotic rectal surgery should not be considered as costs increasing modality unless its effect on total costs of the treatment has been evaluated.

This study has a few limitations. First, the number of patients and operations is too small to do reliable subgroup analyses or compare the costs of three different techniques. Secondly, the Finnish way of calculating operative costs based on procedural code pricing is not applicable in most of the countries. However, scientific studies on surgical cost estimation are methodologically heterogeneous and show little consensus regarding the factors that contribute to operative costs; as a result, the concept of surgical cost remains elusive (PMID: 35191067). Our operative cost estimates are based on the OUH activity-based costing system, which accounts for the aggregated resources utilized for operations within our institution. In addition, our robotic cost estimates include calculated capital and instrument-related expenses associated with robotic procedures. Therefore, the costs presented here are not based on prices paid by patients, county reimbursements, or OUH revenue, but rather represent, as far as possible, the actual resource utilization for each individual patient. The study strengths include the comprehensive and definitive total cost collection of surgical treatment of primary rectal cancer in our financial system. Additionally, the clinical evaluation was based on registry of consecutive patients with a comprehensive access to patient records, offering more complete data coverage compared to registry-based studies, including all surgical and perioperative details and postoperative complications.

## Conclusions

According to our results, robotic and laparoscopic techniques in clinical setting of rectal cancer surgery are not fully substitutional. Instead, patients are allocated to different approaches according to the best clinical assessment leading to skewed distribution of patients. Increasing robotic access changes this patient allocation and increases mini-invasive surgery by replacing some of open rectal resections instead of sole laparoscopic operations. Therefore, cost comparison of robotic and laparoscopic rectal resections may not be a correct method to evaluate financial basis of robotic investment. In our unit, increased operative costs of a new robotic platform were offset by cost reductions in other segments of the treatment. Therefore, increasing robotic surgery may not increase total hospital costs of rectal cancer surgery.

## Data Availability

Data is provided within the manuscript.
